# Novel nomograms for predicting survival for immediate breast reconstruction patients diagnosed with invasive breast cancer—a single-center 15-year experience

**DOI:** 10.3389/fonc.2023.1202650

**Published:** 2023-06-22

**Authors:** Shanshan He, Qingjinan Chen, Gang Li, Bowen Ding, Shu Wang, Chunyong Han, Jingyan Sun, Qingfeng Huang, Jian Yin

**Affiliations:** ^1^ Department of Breast Reconstruction, Tianjin Medical University Cancer Institute and Hospital, Tianjin, China; ^2^ National Clinical Research Center for Cancer, Tianjin Medical University Cancer Institute and Hospital, Tianjin, China; ^3^ Key Laboratory of Cancer Prevention and Therapy, Tianjin Medical University Cancer Institute and Hospital, Tianjin, China; ^4^ Key Laboratory of Breast Cancer Prevention and Therapy, Tianjin Medical University, Ministry of Education, Tianjin, China; ^5^ Tianjin’s Clinical Research Center for Cancer, Sino‐Russian Joint Research Center for Oncoplastic Breast Surgery, Tianjin, China; ^6^ School of Pharmacy, University College London, London, United Kingdom

**Keywords:** survival, invasive breast cancer, immediate breast reconstruction, breast cancer-specific survival, disease-free survival, nomogram

## Abstract

**Background:**

Immediate breast reconstruction is widely accepted following oncologic mastectomy. This study aimed to build a novel nomogram predicting the survival outcome for Chinese patients undergoing immediate reconstruction following mastectomy for invasive breast cancer.

**Methods:**

A retrospective review of all patients undergoing immediate reconstruction following treatment for invasive breast cancer was performed from May 2001 to March 2016. Eligible patients were assigned to a training set or a validation set. Univariate and multivariate Cox proportional hazard regression models were used to select associate variables. Two nomograms were developed based on the training cohort for breast cancer-specific survival (BCSS) and disease-free survival (DFS). Internal and external validations were performed, and the C-index and calibration plots were generated to evaluate the performance (discrimination and accuracy) of the models.

**Results:**

The 10-year estimated BCSS and DFS were 90.80% (95% CI: 87.30%–94.40%) and 78.40% (95% CI: 72.50%–84.70%), respectively, in the training cohort. In the validation cohort, they were and 85.60% (95% CI, 75.90%–96.50%) and 84.10% (95% CI, 77.80%–90.90%), respectively. Ten independent factors were used to build a nomogram for prediction of 1-, 5- and 10-year BCSS, while nine were used for DFS. The C-index was 0.841 for BCSS and 0.737 for DFS in internal validation, and the C-index was 0.782 for BCSS and 0.700 for DFS in external validation. The calibration curve for both BCSS and DFS demonstrated acceptable agreement between the predicted and actual observation in the training and the validation cohorts.

**Conclusion:**

The nomograms provided valuable visualization of factors predicting BCSS and DFS in invasive breast cancer patients with immediate breast reconstruction. The nomograms may have tremendous potential in guiding individualized decision-making for physicians and patients in choosing the optimized treatment methods.

## Introduction

The surgical treatment of breast cancer has evolved immensely since Halsted introduced the first radical mastectomy in 1889 (1). In modern-day practice, nipple-sparing and skin-sparing mastectomy have proven to be oncologically sound and are now widely adopted as the standard of care at many institutions. With the preservation of the skin and in certain circumstances the nipple areolar complex, reconstruction has also witnessed tremendous advancements with improved patient satisfaction and patient-reported outcomes. As such, immediate breast reconstruction has also become an integral component in the multidisciplinary treatment for breast cancer patients. Although scientific studies posed oncological concerns that immediate reconstruction may change regional microenvironment, prolong surgical duration, delay the initiation of subsequent adjuvant therapy, and potentially lead to cancer recurrence, numerous clinical reports were published on the outcomes of immediate breast reconstruction following mastectomy and most supported its long-term oncological safety (2–4) compared to mastectomy alone. Our previous studies have demonstrated similar breast cancer-specific survival (BCSS) and local regional control between immediate implant-based and autologous breast reconstructions (5). The present study aims to investigate risk factors associated with the survival outcomes in breast cancer patients with invasive disease.

A nomogram is a pictorial calculating diagram, invented in 1884 for engineers, to approximate computation of a complex mathematical function. It is a graphical visualization of a complex model of equation and is currently largely utilized in clinical oncology to predict the patients’ outcomes, and therefore aids in the personalized decision making for physicians (6). Individuals are scaled on a series of risk factors and the overall probability is calculated by the nomogram for a specific outcome. Owing to the paucity of established nomograms predicting survival outcomes in breast cancer patients undergoing immediate reconstruction, we aim to develop novel nomograms predicting the probability of long-term BCSS and disease-free survival (DFS) in this specific patient population.

## Patients and methods

### Study population and design

The breast reconstruction database in the National Clinical Research Center in Tianjin Medical University Oncology Hospital was established in 2015 and contained all patients receiving breast reconstructions in the hospital. The database included demographic, disease, and treatment information, and follow-up data including survival, satisfaction, and photographic records. Patients undergoing immediate breast reconstruction were retrospectively collected from the database from May 2001 to March 2016 following Institutional Review Board approval (approval no. 2021103). Patients diagnosed with invasive breast cancer were included. Patients diagnosed with inflammatory breast cancer, stage IV disease, Phyllodes tumor, angiosarcoma, or benign diseases were excluded. Patient demographics, pathological features, treatment approaches, and surgical outcomes were collected. Survival outcomes including locoregional recurrence, distant metastasis, and survival status were also retrieved from the database. The primary outcome of our study was BCSS and DFS. The study was conducted in accordance with the Declaration of Helsinki.

Tumor staging was evaluated according to the American Joint Committee on Cancer (AJCC) pathologic staging. The status of lymph node metastasis, lymphovascular invasion (LVI), soft-tissue invasion, multifocality, and histologic grade was ascertained on pathological assessment. The hormonal receptor was considered negative if less than 1% of cancer cells expressed ER/PR. Because of the unavailability of p53 status, it was excluded for further analysis.

BCSS was defined as the duration from the date of diagnosis until death from breast cancer. DFS referred to the presence of locoregional recurrence or distant metastasis. Locoregional recurrence was defined as recurrence in the ipsilateral chest wall (skin/subcutaneous tissue/muscle), supraclavicular/infraclavicular region, axilla, and/or internal mammary region. All cases of DFS were confirmed either by imaging or by pathological biopsy.

### Statistical analysis

Patients eligible for the analysis were randomly assigned to a training and validation set at a 7:3 ratio. Baseline characteristics were compared between the two sets by Pearson’s chi-square test or Fisher’s exact test for categorical variables. Continuous variables were compared with *t*-test, or Wilcoxon rank-sum test if the Shapiro–Wilks test did not meet the assumption for normality. Univariate and multivariate Cox proportional hazard regression models were used to calculate the association of a series of clinical–pathological factors and BCSS and DFS. Test of proportional hazards assumption with Schoenfeld residuals were performed for the Cox model. Backward selection with Akaike information criterion (AIC) was used to select variables for the multivariate Cox proportional hazard regression models. Hazard ratios (HRs) were presented with 95% confidence intervals (CIs).

Two separate nomograms were constructed based on the results of the Cox regression model. Harrell’s concordance statistics (C-index) were calculated to evaluate the performance of the nomograms. A C-index value of 0.5 indicates the absence of discrimination, whereas 1.0 indicates perfect separation of patients with different outcomes. The accuracy of the nomograms was evaluated visually using the calibration plot with bootstrapping with 1,000 resamples at 5 years and 10 years, respectively. A graphical representation of the relationship between the observed outcome frequencies and the predicted probabilities was produced in the plot, with a 45° diagonal line representing perfect performance of an ideal model.

All analyses were performed using R software (7) (Version 4.0.2, http://www.r-project.org), and the main packages employed included “survival”, “survminer”, “caret”, “MASS”, and “rms”. All tests were two sided, and *p* < 0.05 was considered statistically significant.

## Results

### Establishment of the nomograms

During the study period, 619 cases were diagnosed with invasive breast cancer and received immediate breast reconstruction. All patients were of Chinese origin. The training set included 434 cases, and the patients’ baseline characteristics are listed in **Table 1**. In the training cohort, with a median follow-up of 73.20 months (IQR 54.34–105.53), there were 30 (6.91%) cases of breast cancer-related deaths and 63 (14.52%) cases of breast cancer-related local/distant recurrence. The 5-year and 10-year estimated BCSS were 94.2% (95% CI: 91.9%–96.5%) and 90.8% (95% CI: 87.3%–94.4%), respectively. The 5-year and 10-year estimated DFS were 87.9% (95% CI: 84.7%–91.2%) and 78.4% (95% CI: 72.5%–84.7%), respectively (**Supplementary Figures 1A, B**).

**Table 1:** Baseline characteristics of the invasive breast cancer cohorts with Chinese origin between the training and the validation set.

Variables	Training set (*n* = 434) Number (%)	Validation set (*n* = 185) Number (%)	*p*-value
Categorical			
Family breast cancer history			1.00 ^#^
No	400 (64.6%)	171 (27.6%)	
Yes	34 (5.5%)	14 (2.3%)	
Smoking status			1.00 ^$^
No	432 (69.8%)	184 (29.7%)	
Yes	2 (0.3%)	1 (0.2%)	
Bilateral malignant tumor			0.07 ^#^
No	405 (65.4%)	180 (29.1%)	
Yes	29 (4.7%)	5 (0.8%)	
Pregnancy post-op			0.56 ^$^
No	431(69.6.%)	185 (29.9%)	
Yes	3 (0.5%)	0 (0%)	
Comorbidity			
No	421 (68.0%)	174 (28.1%)	0.13 ^#^
Yes	13 (2.1%)	11 (1.8%)	
Side			0.81 ^#^
Left	219 (35.4%)	96 (15.5%)	
Right	215 (34.7%)	89 (14.4%)	
LVI			0.98 ^#^
No	396 (64.0%)	168 (27.1%)	
Yes	38 (6.1%)	17 (2.7%)	
STI			0.42 ^#^
No	404 (65.3%)	168 (27.1%)	
Yes	30 (4.8%)	17 (2.7%)	
Grade			0.05 ^#^
I	15 (2.4%)	6 (1.0%)	
II	359 (58.0%)	166 (26.8%)	
III	60 (9.7%)	13 (2.1%)	
Multi-focal			0.80 ^#^
No	409 (66.1%)	176 (28.4%)	
Yes	25 (4.0%)	9 (1.5%)	
AJCC stage			0.49 ^#^
I	139 (22.5%)	62 (10.0%)	
II	230 (37.2%)	102 (16.5%)	
III	65 (10.5%)	21 (3.4%)	
Hormonal receptor status			0.10 ^#^
Negative	103 (16.6%)	32 (5.2%)	
Positive	331 (53.5%)	153 (24.7%)	
Her-2			0.29 ^#^
0–1+/FISH(-)	219 (35.4%)	101 (16.3%)	
2+	39 (6.3%)	21 (3.4%)	
3+/FISH(+)	58 (9.4%)	16 (2.6%)	
Unknown	118 (19.1%)	47 (7.6%)	
Ki-67			0.29 ^#^
<15%	58 (9.4%)	23 (3.7%)	
≥15%	234 (37.8%)	112 (18.1%)	
Unknown	142 (22.9%)	50 (8.1%)	
Chemotherapy			0.09 ^#^
None	11 (1.8%)	9 (1.5%)	
Neoadjuvant	50 (8.1%)	18 (2.9%)	
Adjuvant	316 (51.1%)	144 (23.3%)	
Unknown	57 (9.2%)	14 (2.3%)	
Radiation			0.14 ^#^
No	299 (48.3%)	139 (22.5%)	
Yes	135 (21.8%)	46 (7.4%)	
Hormonal therapy			0.23 ^#^
No	116 (18.7%)	42 (6.8%)	
Yes	300 (48.5%)	139 (22.5%)	
Unknown	18 (2.9%)	4 (0.6%)	
Breast surgery type			0.16 ^#^
NSM	146 (23.6%)	66 (10.7%)	
SSM	268 (43.3%)	104 (16.8%)	
BCT	20 (3.2%)	15 (2.4%)	
Axillary surgery			0.82 ^#^
SLNB	61 (9.9%)	24 (3.9%)	
ALND	373 (60.3%)	161 (26.0%)	
Type of reconstruction			0.55 ^#^
Implant-based	198 (32.0%)	90 (14.5%)	
Autologous	236 (38.1%)	95 (15.3%)	
Post-op complications			0.19 ^#^
No	376 (60.7%)	152 (24.6%)	
Yes	58 (9.4%)	33 (5.3%)	
Secondary surgery ^϶^			0.55 ^#^
no	402 (64.9%)	168 (27.1%)	
yes	32 (5.2%)	17 (2.7%)	
Lipo-filling			0.49 ^#^
NO	427 (69.0%)	184 (29.7%)	
YES	7 (1.1%)	1 (0.2%)	
Numerical	Mean ± SD	Mean ± SD	P value
Age (years)	39.8 ± 0.4	40.6 ± 0.5	0.16 ^†^
BMI (kg/m^2^)	22.9 ± 0.1	23.0 ± 0.2	0.77 ^†^
Positive Nodes	1.8 ± 0.2	1.6 ± 0.3	0.41 ^†^
Total nodes	18.1 ± 0.4	18.6 ± 0.6	0.61 ^†^
Number of 2nd surgery	0.1 ± 0.0	0.1 ± 0.0	0.74 ^†^
Number of lipo-filling	0.0 ± 0.0	0.0 ± 0.0	0.28 ^†^

϶ Secondary surgery referred to revision or salvage surgery that were not related to tumor relapse.

LVI, Lymphovascular invasion; STI, Soft tissue invasion; NSM, Nipple-sparing mastectomy; SSM, Skin-sparing mastectomy; BCT, Breast conservation therapy; SLNB, Sentinel lymph node biopsy; ALND, Axillary lymph node dissection; BMI, Body mass index.

# Pearson’s chi-square test.

$ Fisher’s exact test.

† Wilcoxon rank-sum test.

Univariate analysis associated with BCSS and DFS demonstrated a number of significant findings (**Table 2**). Breast cancer with positive LVI (HR 3.8, 95% CI: 1.70–8.50, *p* = 0.001), more axillary lymph node involvement (HR 1.10, 95% CI: 1.00–1.10, *p* < 0.00001), advanced tumor stage (HR 10.80, 95% CI: 3.10–37.60, *p* = 0.04), receiving autologous breast reconstruction (HR 2.50, 95% CI: 1.00–5.70, *p* = 0.04), and adjuvant radiation (HR 6.70, 95% CI: 3.00–15.00, *p* < 0.00001) were associated with worse breast cancer-related survival. Variables associated with worse DFS were younger age at diagnosis (HR 0.96, 95% CI: 0.93–0.99, *p* = 0.02), post-operative pregnancy (HR 5.10, 95% CI: 1.20–21.00, *p* = 0.024), LVI (HR 2.80, 95% CI: 1.50–5.20, *p* = 0.0014), more axillary lymph node involvement (HR 1.10, 95% CI: 1.00–1.10, *p* < 0.00001), stage II and III disease (stage II vs stage I; HR 2.14, CI 95%: 1.02–4.47, *p* = 0.04; stage III vs stage I; HR 5.95, 95% CI: 2.72–12.01, *p* < 0.00001), and adjuvant radiation (HR 2.80, 95% CI 1.70–4.60, *p* < 0.0001). The graphical diagnostic of the Schoenfeld residuals against the transformed time was performed, and the global test was insignificant for both Cox models for BCSS and DFS (*p* = 0.1327, *p* = 0.1442, **Supplementary Figures 2A, B**).

**Table 2:** Characteristics associated with BCSS and DFS by univariate analysis in patients in the training set.

Univariate Analysis	BCCS	*p*-value	DFS	*p*-value
	HR (95% CI)		HR (95% CI)	
Categorical variables				
Family breast cancer history	0.75 (0.18–3.10)	0.69	0.74 (0.27–2.00)	0.57
Comorbidities	1.90 (0.25–14.00)	0.53	1.90 (0.46–7.80)	0.28
Smoking status	3*10^−7^ (0.00–Inf)	1.00	3*10^−7^ (0.00–Inf)	1.00
Bilateral malignant tumor	2.40 (0.85–7.00)	0.10	1.90 (0.88–4.20)	0.10
Pregnancy post-op	5.00 (0.68–37.00)	0.11	5.10 (1.20–21.00)	0.024^*^
Side (right vs. left)	1.4 (0.68–2.90)	0.37	1.10 (0.67–1.80)	0.72
LVI	3.8 (1.70–8.50)	0.001^*^	2.80 (1.50–5.20)	0.0014^*^
STI	2.1 (0.73–6.00)	0.17	1.80 (0.82–3.90)	0.15
Grade				
I	1.00		1.00	
II	0.87 (0.12–6.48)	0.89	1.04 (0.25–4.29)	0.95
III	1.80 (0.22–14.65)	0.58	1.51 (0.33–6.80)	0.59
Multifocal breast cancer	1.30 (0.32–5.6)	0.69	1.70 (0.68–4.30)	0.26
AJCC stage				
I	1.00		1.00	
II	2.42 (0.66–8.53)	0.17	2.14 (1.02–4.47)	0.04^*^
III	10.80 (3.10–37.60)	0.0002^*^	5.95 (2.72–12.01)	<0.00001^*^
Hormonal receptor (positive vs. negative)	0.77 (0.35–1.70)	0.52	0.88 (0.50–1.50)	0.65
Her-2 Status				
0–1+/FISH(−)	1.00		1.00	
2+	2.28 (0.81–6.38)	0.12	0.97 (0.41–2.30)	0.94
3+/FISH(+)	2.19 (0.87–5.48)	0.10	1.30 (0.66–2.56)	0.45
Unknown	0.55 (0.17–1.67)	0.29	0.50 (0.25–1.01)	0.05
Ki-67				
<15%	1.00		1.00	
≥15%	3.06 (0.40–23.38)	0.28	1.53 (0.59–3.93)	0.38
Unknown	4.81 (0.63–36.56)	0.13	1.64 (0.63–4.29)	0.32
Chemotherapy				
None	1.00		1.00	
Neoadjuvant	2.51×10^7^ (0–Inf)	1.00	1.32×10^7^ (0–Inf)	1.00
Adjuvant	1.05×10^7^ (0–Inf)	1.00	1.07×10^7^ (0–Inf)	1.00
Unknown	2.86×10^6^ (0–Inf)	1.00	2.30×10^7^ (0–Inf)	1.00
Radiation	6.7 (3.00–15.00)	<0.00001^*^	2.80 (1.70–4.60)	<0.0001^*^
Hormonal therapy				
No	1.00		1.00	
Yes	0.97 (0.45–2.13)	0.95	1.19 (0.67–2.10)	0.55
Unknown	3.80×10^−8^ (0–Inf)	0.10	0.41 (0.05–3.11)	0.39
Type of breast resection				
NSM	1.0		1.0	
SSM	1.47 (0.65–3.33)	0.35	1.36 (0.79–2.36)	0.27
BCT	0.71 (0.09–5.75)	0.75	0.64 (0.15–2.76)	0.55
Axillary surgery (ALND vs. SLNB)	3.6 (0.49–27.00)	0.21	1.40 (0.57 –3.60)	0.44
Type of reconstruction (autologous vs. implant-based)	2.50 (1.00–5.70)	0.04^*^	1.2 (0.27–2.00)	0.44
Post-op complications	1.70 (0.72–4.00)	0.23	1.50 (0.85–2.80)	0.15
Secondary surgery ^϶^	0.87 (0.21–3.60)	0.85	0.59 (0.19–1.90)	0.37
Lipo-filling	1.10×10^−7^ (0–Inf)	1.00	1.10×10^−7^ (0–Inf)	1.00
Continuous variables				
Age	1.00 (0.96–1.10)	0.88	0.96 (0.93–0.99)	0.02^*^
BMI (kg/m^2^)	1.10 (0.98–1.30)	0.10	1.10 (0.96–1.10)	0.27
Number of positive lymph nodes	1.10 (1.00–1.10)	<0.00001^*^	1.10 (1.00–1.10)	<0.00001^*^
Number of total lymph nodes	1.00 (0.99–1.10)	0.12	1.00 (0.99–1.10)	0.11
Number of secondary surgery	0.80 (0.23–2.80)	0.72	0.74 (0.30–1.80)	0.51
Number of lipofilling	1.90×10^−7^ (0–Inf)	1.00	2.00×10^−7^ (0–Inf)	0.99

϶ Secondary surgery referred to revision or salvage surgery that were not related to tumor relapse.

LVI, Lymphovascular invasion; STI, Soft tissue invasion; Inf, Infinity; NSM, Nipple-sparing mastectomy; SSM, Skin-sparing mastectomy; BCT, Breast conservation therapy; SLNB, Sentinel lymph node biopsy; ALND, Axillary lymph node dissection; BMI, Body mass index.

*p < 0.05.

After backward selection with AIC (**Table 3**), variables associated with worse BCSS on multivariate analysis were bilateral breast malignancy (HR 8.17, 95% CI: 2.38–28.08, *p* < 0.001), adjuvant radiation (HR 5.85, 95% CI: 1.92–17.76, *p* = 0.002), and LVI (HR 2.57, 95% CI: 1.03–6.40, *p* = 0.04). Variables associated with worse DFS on the multivariate analysis were younger age (HR 0.96, 95% CI: 0.93–0.999, *p* = 0.04), comorbid medical illnesses (HR 7.13, 95% CI: 1.49–34.06, *p* = 0.01), bilateral disease (HR 3.43, 95% CI: 1.51–7.80, *p* = 0.003), advanced stage (stage III vs stage I; HR 3.12, 95% CI: 1.14–8.52, *p* = 0.03), and number of positive axillary nodes (HR 1.05, 95% CI: 1.004–1.11, *p* = 0.03) (**Table 4**).

**Table 3:** Characteristics associated with BCSS and DFS in patients in the training set with variables selected using backward stepwise selection with the Akaike information criterion.

Backward selection with least AIC	BCCS		Backward selection with least AIC	DFS	*p*-value
	HR (95% CI)	*p*-value		HR (95% CI)	
*Variables selected*			*Variables selected*		
Comorbidities	9.07 (1.06–77.41)	0.04^*^	Comorbidities	7.93 (1.68–37.48)	0.009^*^
Bilateral malignant tumor	6.83 (2.10–22.54)	0.001^*^	Bilateral malignant tumor	3.19 (1.41–7.18)	0.005^*^
Side	1.83 (0.86–3.87)	0.12	Age	9.53 (0.92–0.99)	0.008^*^
Type of breast resection (autologous vs. implant-based)	1.92 (0.76–4.88)	0.17	Positive nodes number	1.06 (1.02–1.11)	0.009^*^
NSM	1.00		Multi-focal	2.69(1.04–6.96)	0.04^*^
SSM	1.58 (0.68–3.66)	0.29	AJCC Stage		
BCT	0.15 (0.01–1.67)	0.12	I	1.00	
Radiation	12.61 (4.91–32.38)	<0.0001^*^	II	2.36 (1.10–5.04)	0.03^*^
Her-2 Status			III	4.14 (1.64–10.45)	0.003^*^
0–1+/FISH(−)	1.00		Hormonal receptor (positive vs. negative)	0.34 (0.11–1.06)	0.06
2+	3.04 (1.03–8.97)	0.04^*^	Hormonal therapy		
3+/FISH(+)	2.48 (0.94–6.53)	0.06	No	1.00	
Unknown	0.62 (0.19–1.99)	0.42	Yes	3.12 (0.99–9.78)	0.051
Ki-67			Unknown	7.51 (0.07–7.95)	0.81
<15%	1.00		Lipo-filling	5.16×10^–8^ (0–Inf)	1.00
≥15%	1.94 (0.25–15.21)	0.53			
Unknown	5.75 (0.73–45.22)	0.96			

NSM, Nipple-sparing mastectomy; SSM, Skin-sparing mastectomy; BCT, Breast conservation therapy; Inf, Infinity. *p < 0.05.

**Table 4:** Characteristics associated with BCSS and DFS by multivariate analysis in patients in the training set.

Multivariate Analysis	BCCS	*p*-value	Multivariate Analysis	DFS	*p*-value
	HR (95% CI)			HR (95% CI)	
BMI (kg/m^2^)	1.08 (0.94–1.24)	0.27	Age	0.96 (0.93–0.999)	0.04^*^
Comorbidities	7.65 (0.85–68.61)	0.07	Comorbidities	7.13 (1.49–34.06)	0.01^*^
Side	1.66 (0.74–3.73)	0.21	Multi-focal	2.51 (0.93–6.78)	0.07
Bilateral malignant tumor	6.00 (1.87–19.27)	0.003^*^	Bilateral malignant tumor	3.43 (1.51–7.80)	0.003^*^
Pregnancy post-op	4.95 (0.56–43.89)	0.15	Pregnancy post-op	3.56 (0.78–16.32)	0.10
Grade			Hormonal receptor	3.45 (0.11–1.10)	0.07
I	1.00		Grade		
II	0.86 (0.11–6.93)	0.89	I	1.00	
III	1.49 (0.18–12.49)	0.71	II	1.08 (0.26–4.51)	0.91
AJCC stage			III	1.18 (0.26–5.40)	0.83
I	1.00		AJCC stage		
II	1.13 (0.28–4.53)	0.86	I	1.00	
III	2.03 (0.43–9.59)	0.37	II	12.10 (0.97–4.55)	0.06
Number of positive nodes	1.02 (0.93–1.10)	0.59	III	3.12 (1.14–8.52)	0.03^*^
LVI	2.57 (1.03–6.40)	0.04^*^	Number of positive nodes	1.05 (1.004–1.11)	0.03^*^
Her-2 status			LVI	1.54 (0.78–3.08)	0.22
0–1+/FISH(−)	1.00		Lipo-filling	1.26×10^−7^ (0–Inf)	0.99
2+	1.77 (0.57–5.54)	0.33	Radiation	1.42 (0.73–2.77)	0.30
3+/FISH(+)	2.03 (0.68–6.06)	0.20	Hormonal therapy		
Unknown	0.47 (0.14–1.56)	0.22	No	1.00	
Ki-67			Yes	2.92 (0.91–9.46)	0.07
<15%	1.00		Unknown	9.23 (0.09–9.84)	0.95
≥15%	1.61 (0.20–13.20)	0.65			
Unknown	3.82 (0.47–30.97)	0.21			
Radiation	4.78 (1.67–13.68)	0.004^*^			
Type of reconstruction (autologous vs. implant-based)	1.70 (0.65– 4.42)	0.27			

BMI, Body mass index; LVI, Lymphovascular invasion; Inf, Infinity. *p < 0.05.

**Figure 1:**
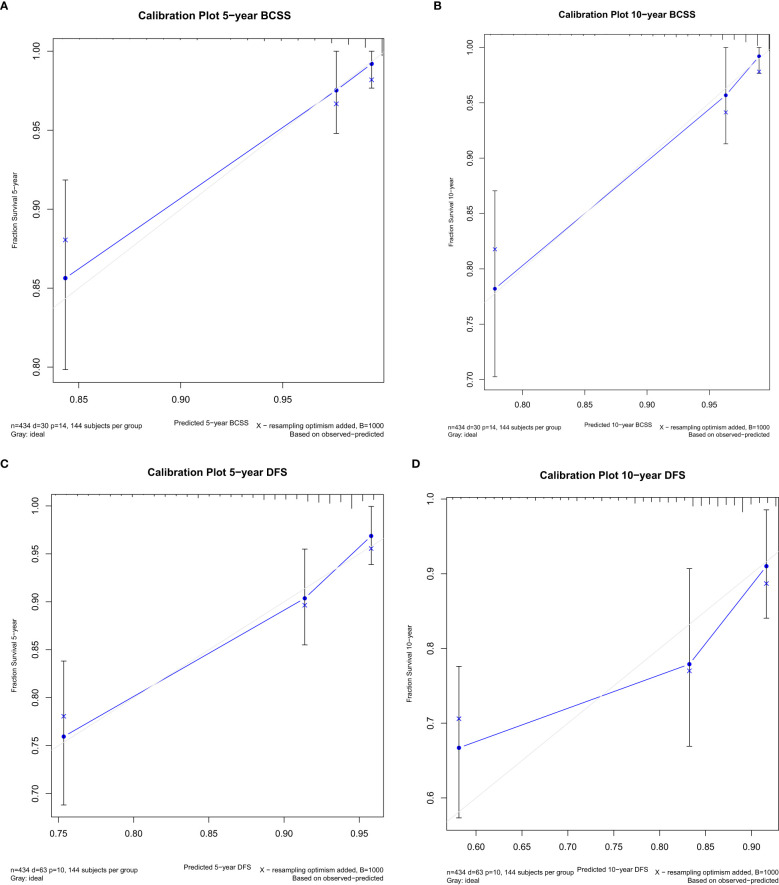
Internal calibration plot at 5 years and 10 years post-op for breast cancer-specific survival (BCSS) **(A, B)** and disease-free survival (DFS) **(C, D)**.

**Figure 2:**
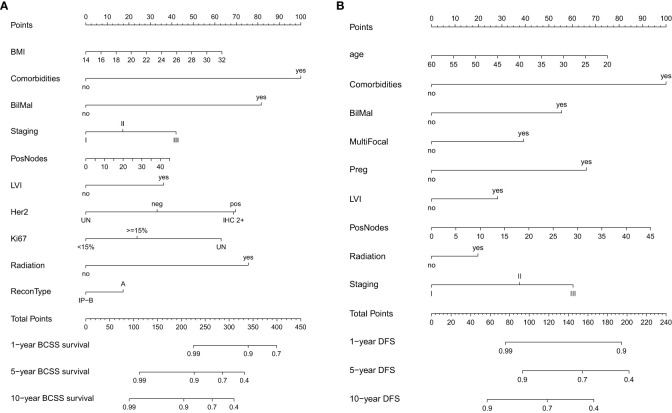
Nomogram for **(A)** breast cancer-specific survival (BCSS) and **(B)** disease-free survival (DFS).

Variables that were significant in univariate analysis, multivariate analysis, and backward stepwise regression, and clinical variables [Ki67, BMI (body mass index)] (8, 9) that were reported to have predicative potentials for survivals were included in the final model for the establishment of the nomogram for BCSS and DFS. For BCSS, the model showed good internal discrimination with a Harrell’s concordance C-index of 0.84. Calibration plot on bootstrap replicate (*B* = 1,000) demonstrated that the model was well calibrated at 5 years and 10 years (**Figures 1A, B**). For DFS, the model showed acceptable internal discrimination with a Harrell’s concordance C-index of 0.74, and the calibration plot showed that the model was well calibrated at 5 years and 10 years (**Figures 1C, D**). Each of the two final models was used to build a nomogram for the prediction of BCSS and DFS at 1-year, 5-year and 10-year post-op (**Figures 2A, B**).

### Validation of the nomograms

The validation cohort included 185 cases of invasive breast cancer who received immediate breast reconstruction. The patients’ baseline characteristics were compared between the two sets and no significant statistical differences were found (**Table 1**).

With a median follow-up of 69.6 months [interquartile range (IQR) 53.8–96.3], there were 14 (7.6%) cases of breast cancer-related death and 24 (13.0%) cases of breast cancer-related local/distant recurrence. The 5-year and 10-year estimated BCSS were 93.5% (95% CI: 89.9%–97.3%) and 85.6% (95% CI: 75.9%–96.5%), respectively. The 5-year and 10-year estimated DFS were 88.8% (95% CI: 84.2%–93.7%) and 84.10% (95% CI: 77.8%–90.9%), respectively (**Supplementary Figures 1C, D**). The BCSS and DFS were similar between the training and the validation cohort (*p* = 0.7, *p* = 0.7).

**Figure 3:**
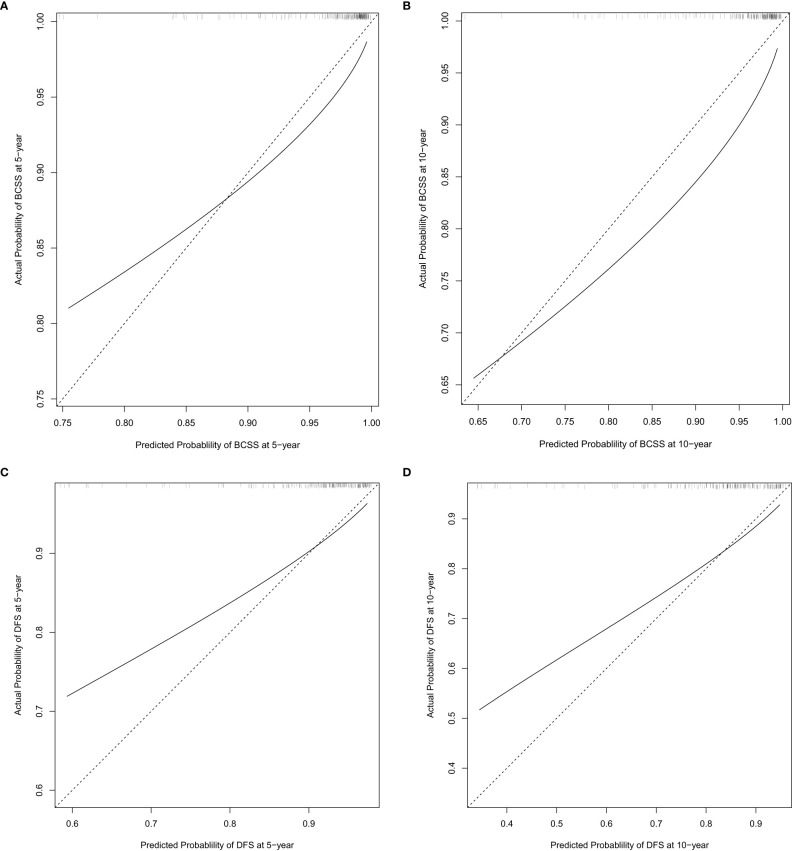
External calibration plot at 5 years and 10 years post-op for breast cancer-specific survival (BCSS) **(A, B)** and disease-free survival (DFS) **(C, D)**.

The nomograms established in the training set were further tested in the validation cohort. Both nomograms showed acceptable performance with a C-index of 0.78 for BCSS and 0.70 for DFS. The predicted 5-year and 10-year probability of BCSS and DFS against the observed probability was plotted and showed acceptable calibration (**Figures 3A–D**).

## Discussion

Breast reconstruction has become an integral component in the comprehensive treatment for female breast cancer patients worldwide. One of the questions that plague breast cancer patients the most is their prognosis and survival after breast reconstruction. However, each patient has individual concerns, especially in forming their decisions as to whether to have immediate breast reconstruction or not. Nomograms were established to estimate the individual risk based on a series of multiple variables and are frequently used in the prediction of lymph node status in breast cancer (10, 11).

Several nomograms were developed in recent years predicting the survival or recurrence for breast cancer patients (12–14); however, no nomograms were established for patients receiving immediate reconstruction. Although, clinically, no differences in oncological outcomes were reported when comparing patients receiving modified radical mastectomy (MRM) with patients undergoing MRM with immediate reconstruction (15, 16), considerable debate exists whether complications following reconstruction may delay adjuvant therapies that can have a negative impact on oncologic outcomes. We have previously demonstrated no differences in oncological outcomes comparing implant-based and autologous breast reconstruction in a propensity score-matched setting (5). The present study expands on our previous work demonstrating factors that can compromise BCSS and DFS in patients who have undergone immediate reconstruction, and also allowed construction and validation of novel nomograms to predict individual BCSS and DFS.

The development of adjuvant treatment such as chemotherapeutic agents and radiation, hormonal, and targeted therapy had significant benefits to the survival of breast cancer patients. In different reports, the 5-year overall survival was between 88% and 94% (15, 17) while the 5-year DFS was between 92% and 95.2% in breast cancer patients receiving immediate breast reconstruction (2, 15, 18). The individual risk of breast cancer-related death and disease progression can be predicted by a combination of clinical–pathological variables using nomograms, which has tremendous potential during patient consultation presenting for reconstruction.

The present study confirms LVI and bilateral breast malignancy as independent risk factors associated with worse BCSS. Contrary to another established nomogram for luminal breast cancer (14) using the Surveillance, Epidemiology, and End Results (SEER) database, which demonstrated that adjuvant radiation was beneficial and protective for BCSS, we demonstrate that adjuvant radiation was associated with worse BCSS. While the findings are contradictory, one would suspect that adjuvant radiation portends a worse prognosis since the need for adjuvant radiotherapy is often a surrogate indicator of more advanced disease. The difference may be attributed to the differences in the target cohort selection and the differences in the indication for local radiation therapy. However, nonetheless, multivariate analysis did not demonstrate any associations between BCSS and the type of immediate breast reconstruction or the occurrence of reconstruction-related complications, which further supported our previous findings that the choice of immediate breast reconstruction did not exert an adverse impact on the long-term survival. Furthermore, the nomogram formulated from the dataset demonstrated a strong correlation index confirming the validity of the nomogram.

In immediate implant-based (19) and autologous (20) breast reconstruction studies, LVI, tumor staging, multifocality, and absence of hormonal therapy were risk factors associated with local regional recurrence. The novel nomogram further confirmed that younger age, comorbidities, advanced tumor staging, bilateral malignancy, and higher number of positive lymph nodes were associated with worse DFS. Of note, neither the type of breast reconstruction nor the occurrence of reconstruction-related complications was associated with inferior local or distant control. Other nomograms demonstrated that young age, more advanced disease, and histologic factors were associated with increased risks of local breast cancer recurrence (12). Unfortunately, the unavailability of the exact Ki-67 and Her-2 status in the earlier data is a significant limitation to the present study. Certainly, adding more molecular subtyping such as p53 and subsequent treatment variables will improve the performance and calibration of our nomogram and is an area of active investigation for future studies.

Our current study presented two nomograms identifying important variables to predict the likelihood of survival for an individual breast cancer patient undergoing immediate reconstruction. To our knowledge, they were the first nomograms established for the reconstruction cohorts. They provided potentials to be integrated into a dynamic outcome calculator during the multidisciplinary treatment for breast cancer patients, and benefited both the patient and the physician in personalized decision-making.

The main limitation of the study was the retrospective design and the lack of data, especially biomarkers such as Her2/FISH and Ki67 in our early cases, as well as the genetic testing results, which required the models to undergo further refinement. Furthermore, this was a single-center study that did not include true external validating data from other institutions; thus, the results required future modifications and may not be directly applicable to other centers. Future studies involving comprehensive and complete biomolecular data, multiple centers, and prospective validation of the nomogram and its derived personalized prediction tool are warranted, and clinical expertise, experience, and a multi-disciplinary approach are still critical for optimizing the care of patients with invasive breast cancer.

## Conclusions

Two nomograms for predicting BCSS and DFS demonstrate good performance and validity and can provide individualized estimates for oncological outcomes and survival that can enhance clinical evaluation, counseling, and management of expectations in patients with invasive disease seeking breast reconstruction.

## Data availability statement

Due to patient confidentiality, data were not open to public and could be made available upon request with permission from the hospital’s ethical board. Requests to access the datasets should be directed to ec_tjcih@126.com.

## Ethics statement

The studies involving human participants were reviewed and approved by Tianjin Medical University Ethical Board. The patients/participants provided their written informed consent to participate in this study.

## Author contributions

Conception and design: SH and JY. Provision of study materials or patients: JY, JS, and QH. Collection and assembly of data: SH, QC, BD, SW, and CH. Data analysis and interpretation: SH and GL. Manuscript writing: SH and JY. All authors contributed to the article and approved the submitted version.
