# Supplementary material for: Novel nomograms for predicting survival for immediate breast reconstruction patients diagnosed with invasive breast cancer—a single-center 15-year experience
Source: Front Oncol. 2023 Jun 22;13:1202650. doi: 10.3389/fonc.2023.1202650 (PMC10325653; doi:10.3389/fonc.2023.1202650)
Supplement: Supplementary file 1 [file DataSheet_1.docx]

**Title**: Novel nomograms for predicting survival for immediate breast reconstruction patients diagnosed with invasive breast cancer- a single center 15-year experience

**Authors**: Shanshan **He** M.D. , Qingjinan **Chen** M.M. , Gang **Li** Ph.D. , Bowen **Ding** M.D. , Shu **Wang** M.D. ,Chunyong **Han** M.D. , Jingyan **Sun** M.D. , Qingfeng **Huang** M.M. , Jian **Yin** M.D.

**Corresponding author**: Jian Yin. [yinjian@tjmuch.com](mailto:yinjian@tjmuch.com)

**Supplementary Figure 1.**

BCSS curve (A, B) and DFS curve (C, D) in the training and validation set.


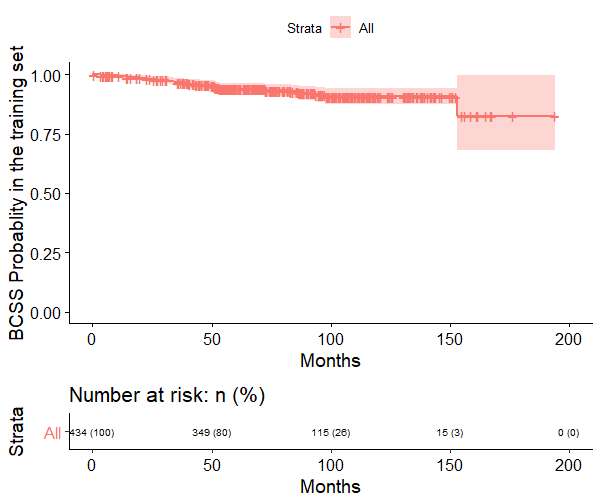

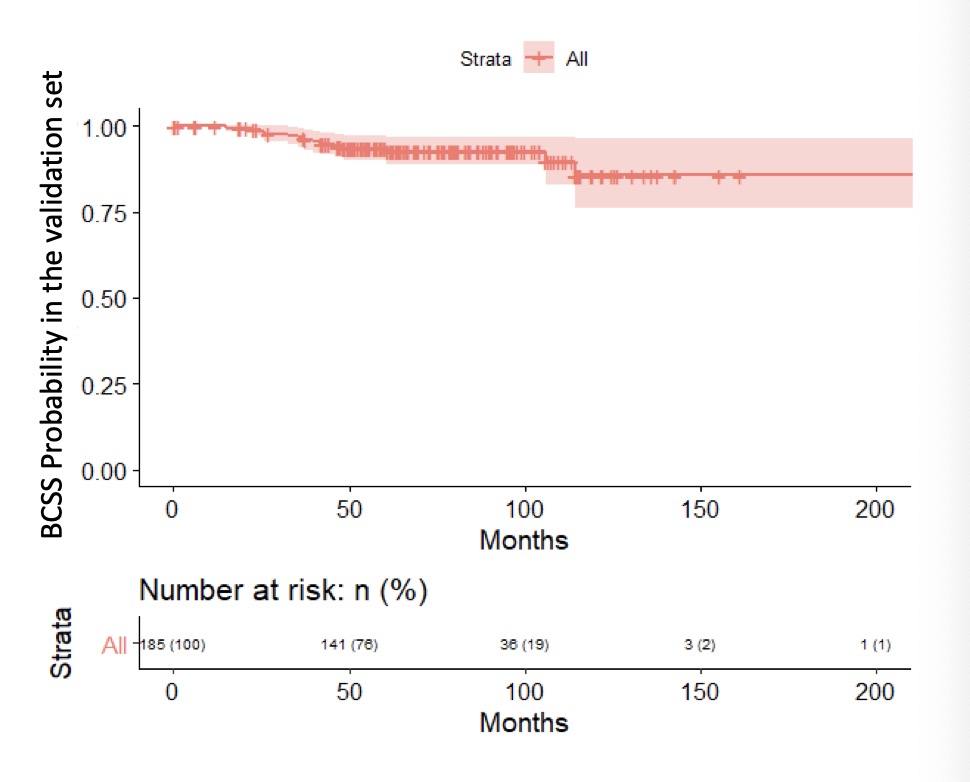


A B


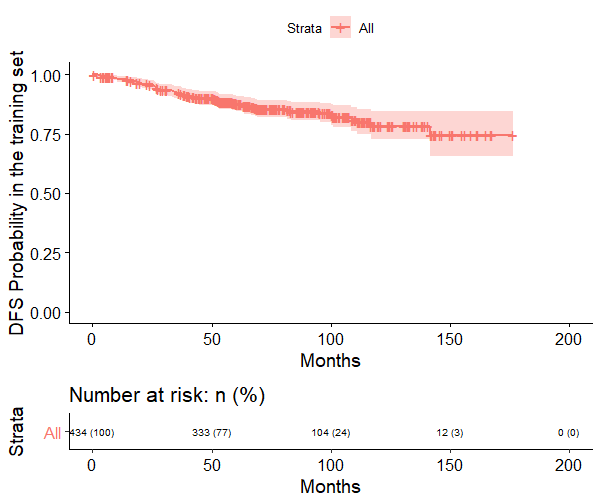

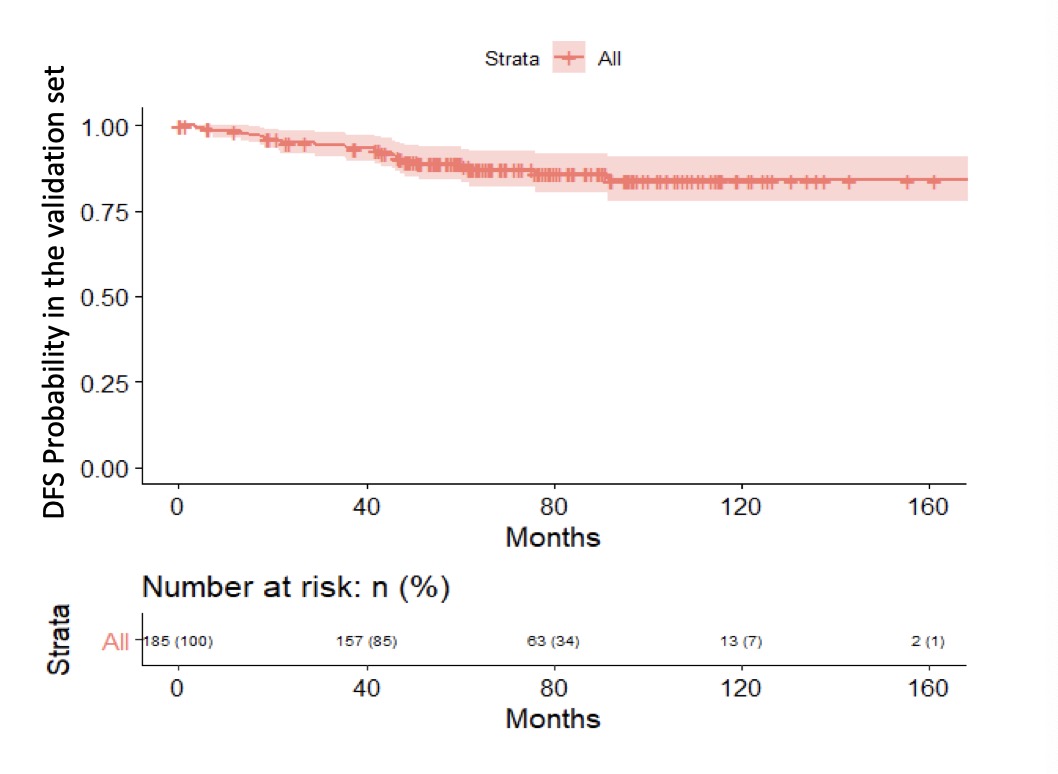


C D

**Supplementary Figure 2.**

Test of proportional hazard (PH) assumption for the cox regression model for BCSS(A) and DFS(B).


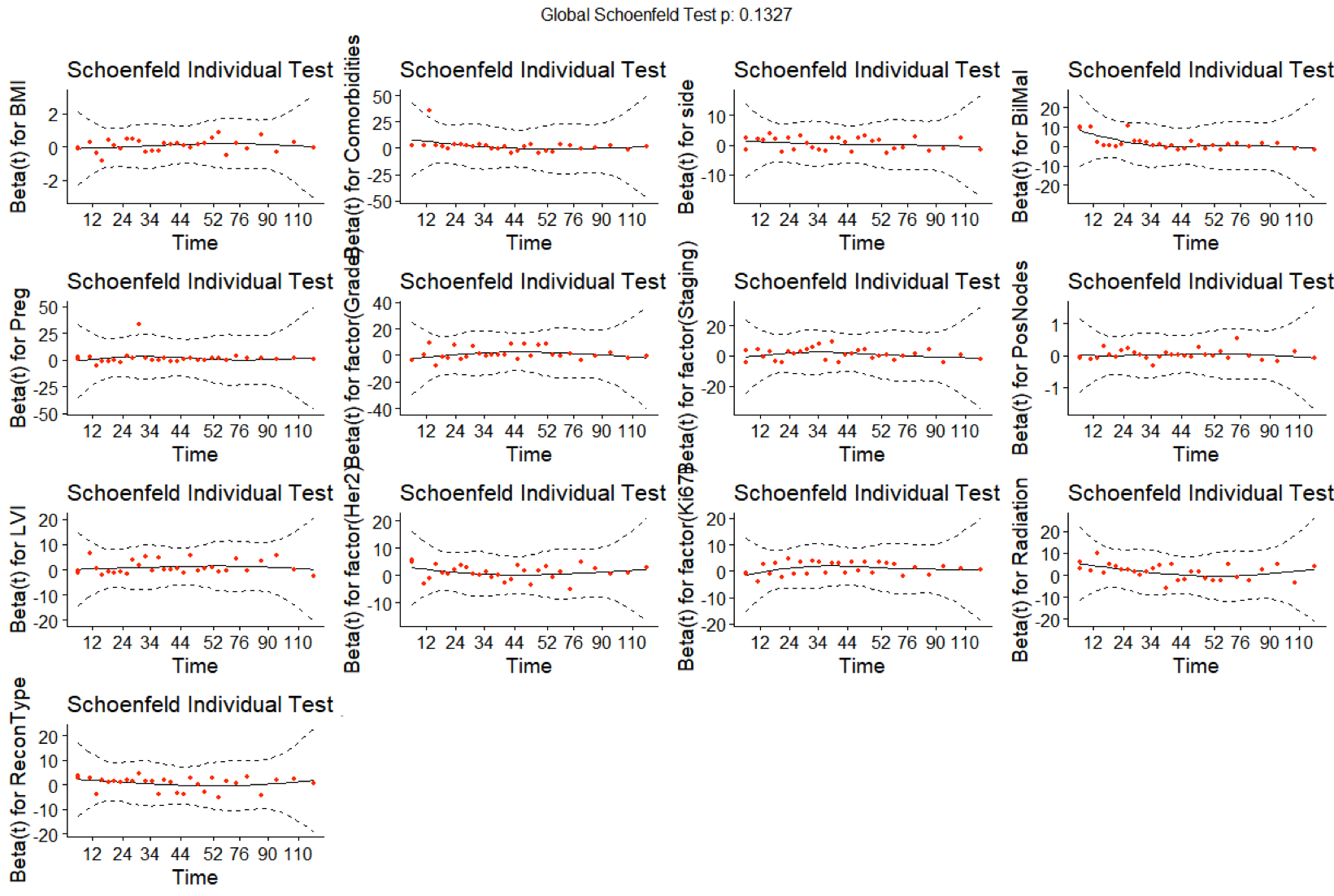


A


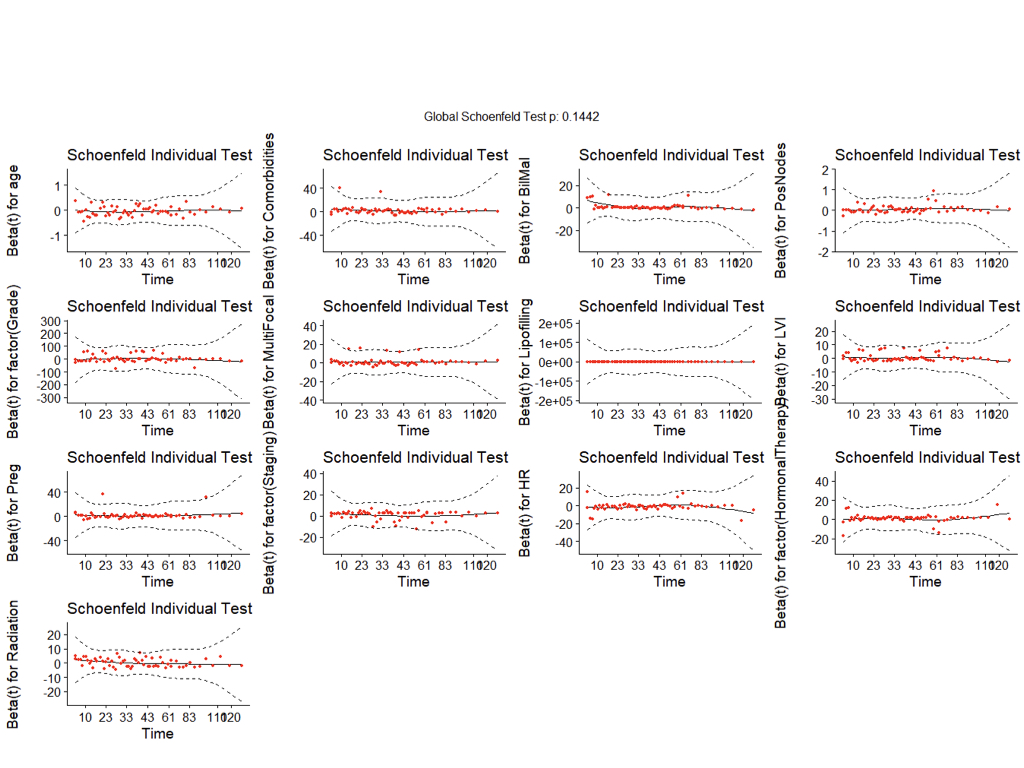


B

**Supplementary Table 1**. Characteristics associated with BCSS and DFS in patients in the training set with variables selected using backward stepwise selection with the Akaike information criterion.

| Backward Selection with least AIC | BCCS |  | Backward Selection with least AIC | DFS |  |
| --- | --- | --- | --- | --- | --- |
|  | HR (95% CI) | P value |  | HR (95% CI) | P value |
| *Variables selected* |  |  | *Variables selected* |  |  |
| Comorbidities | 9.07 (1.06-77.41) | 0.04^*^ | Comorbidities | 7.93 (1.68-37.48) | 0.009^*^ |
| Bilateral malignant tumor | 6.83 (2.10-22.54) | 0.001^*^ | Bilateral malignant tumor | 3.19 (1.41-7.18) | 0.005^*^ |
| Side | 1.83 (0.86-3.87) | 0.12 | Age | 9.53 (0.92-0.99) | 0.008^*^ |
| Type of breast resection (autologous vs. implant-based) | 1.92 (0.76-4.88) | 0.17 | Positive nodes number | 1.06 (1.02-1.11) | 0.009^*^ |
| NSM | 1.00 |  | Multi-focal | 2.69(1.04-6.96) | 0.04^*^ |
| SSM | 1.58 (0.68-3.66) | 0.29 | AJCC Stage |  |  |
| BCT | 0.15 (0.01-1.67) | 0.12 | I | 1.00 |  |
| Radiation | 12.61 (4.91-32.38) | <0.0001^*^ | II | 2.36 (1.10-5.04) | 0.03^*^ |
| Her-2 Status |  |  | III | 4.14 (1.64-10.45) | 0.003^*^ |
| 0-1+/FISH(-) | 1.00 |  | Hormonal receptor  (positive vs. negative) | 0.34 (0.11-1.06) | 0.06 |
| 2+ | 3.04 (1.03-8.97) | 0.04^*^ | Hormonal therapy |  |  |
| 3+/FISH(+) | 2.48 (0.94-6.53) | 0.06 | no | 1.00 |  |
| unknown | 0.62 (0.19-1.99) | 0.42 | yes | 3.12 (0.99-9.78) | 0.051 |
| Ki-67 |  |  | unknown | 7.51 (0.07-7.95) | 0.81 |
| <15% | 1.00 |  | Lipo-filling | 5.16×10^-8^ (0-Inf) | 1.00 |
| ≥15% | 1.94 (0.25-15.21) | 0.53 |  |  |  |
| unknown | 5.75 (0.73-45.22) | 0.96 |  |  |  |

NSM= Nipple sparing mastectomy

SSM= Skin sparing mastectomy

BCT=Breast conservation therapy

Inf=Infinity

* P<0.05
